# Food insecurity among Asian Americans: A scoping review protocol

**DOI:** 10.1371/journal.pone.0287895

**Published:** 2023-07-03

**Authors:** Suji Ro, Nhat-Ha Pham, Victoria N. Huynh, Q. Eileen Wafford, Milkie Vu

**Affiliations:** 1 Weinberg College of Arts and Sciences, Northwestern University, Evanston, Illinois, United States of America; 2 College of Arts and Sciences, University of Pennsylvania, Philadelphia, Pennsylvania, United States of America; 3 Emory College of Arts & Sciences, Emory University, Atlanta, Georgia, United States of America; 4 Galter Health Sciences Library & Learning Center, Feinberg School of Medicine, Northwestern University, Chicago, Illinois, United States of America; 5 Department of Preventive Medicine, Feinberg School of Medicine, Northwestern University, Chicago, Illinois, United States of America; Xiamen University - Malaysia Campus: Xiamen University - Malaysia, MALAYSIA

## Abstract

**Introduction:**

Food insecurity is prevalent in the U.S. and is associated with deleterious health, behavioral, and social consequences. Food insecurity is currently addressed largely through public and private food assistance programs (e.g., the Supplementary Nutrition Assistance Program, and food pantries). A body of research has explored racial and ethnic disparities and differences in food insecurity and coping strategies. However, limited literature has explored these experiences among Asian Americans and Asian origin groups in the United States.

**Objective:**

The aim of this review is to establish what is known about the experience of food insecurity and nutrition program participation in the Asian American population and among Asian origin groups and to suggest further research and policy action to better address food insecurity in this population.

**Methods:**

Our review is guided by the methodological framework proposed by Arksey and O’Malley and refined and outlined by Levac and colleagues and the Joanna Briggs Institute. We will search key terms related to food insecurity and Asian Americans in Medline (Ovid), the Cochrane Library (Wiley), CINAHL Plus with Full Text (Ebsco), PsycINFO (Ebsco), and Scopus (Elsevier). An article will be included if it was published in the English language; is a peer reviewed research manuscript and reports primary research findings from analyses; and describes food insecurity or strategies to cope with food insecurity among individuals of Asian origins living in the U.S. An article will be excluded if it is a book, conference proceedings, or grey literature (e.g., thesis or dissertation); is a commentary, editorial, or opinion piece without primary research data; contains only research conducted outside of the U.S.; includes Asians in the sample but does not provide separate data on food insecurity or strategies to cope with food insecurity among Asians; and describes only dietary changes or patterns but not food insecurity. Two or more reviewers will participate in the study screening and selection process. We will record information from the final articles chosen to be included in the review in a data table template and will also prepare a summary narrative with key findings.

**Expected outputs:**

Results will be disseminated through peer-reviewed publications and conference presentations. The findings from this review will be of interest to researchers and practitioners and inform further research and policy to better address food insecurity among this population.

## Introduction

Food insecurity can be generally understood as “the limited or uncertain availability of nutritionally adequate and safe foods” or “limited or uncertain ability to acquire acceptable foods in socially acceptable ways” [1]. More than 13.8 million households in the United States (U.S.) currently struggle with food insecurity [2], with “their access to adequate food for active, healthy living limited by lack of money and other resources” [3]. A 2021 report from the U.S. Department of Agriculture found that 20% of non-Hispanic Black households, 16% of Hispanic households, and 7% of non-Hispanic White households struggled with food insecurity (no separate data was provided for non-Hispanic Asian households) [4].

The prevalence of food insecurity in the U.S. is a serious cause for concern. Its many adverse health consequences include increased rates of diabetes, hypertension, and poor sleep [5]. Among Asian American women and children in particular, food insecurity has been found to be a predictor of obesity, anemia, and overall general worse health [6–8]. Food insecurity is also associated with increased behavioral and mental health complications, such as aggression and anxiety in children [9] and depression in adults [10, 11]. Furthermore, these outcomes have broader societal consequences. One such impact is higher healthcare utilization and cost. Households that experience food insecurity have significantly higher healthcare utilization such as emergency department visits and inpatient admissions [12, 13]. Similarly, food insecurity increases the cost of public education and emergency food distribution and lowers worker productivity, leading to a total associated cost of more than $167.5 billion in the U.S. annually [14]. Prevalence of food insecurity is influenced by a variety of factors, including individual- and group-level factors as well as broader environmental factors, which indicates that populations in certain areas are more vulnerable to food insecurity. Examples of broader environmental factors that increase vulnerability to food insecurity include climate change, disease epidemics and pandemics, and war; these factors are especially important to consider when developing, recommending, and implementing food insecurity coping strategies [15–17].

Coping strategies for food insecurity currently largely involve public and private food assistance programs. The Supplemental Nutrition Assistance Program (SNAP) is the largest public assistance program in the U.S. and assists 38 million people each year [18]. The eligibility for SNAP is determined by states and is based on resource and income levels. Eligible households receive monthly allotments that can be used to buy groceries at authorized food retailers [19]. Other nutrition assistance programs such as the Special Supplemental Nutrition Program for Women, Infants, and Children (WIC) aid especially vulnerable populations. WIC currently reaches around half of all infants born in the U.S. and provides checks or vouchers for food purchases as well as nutrition education and referrals to services [20]. Additionally, private food assistance also plays an important role. For example, food pantry networks, comprised of more than 60,000 pantries, reach up to 4.4% of the U.S. population and help alleviate the burden of food insecurity for many families [21, 22]. These assistance programs, however, are still often underutilized due to barriers to participation such as stigma, complex procedures, and lack of knowledge about eligibility [23–25]. As participation in these programs improves outcomes in dietary intake, various non-health outcomes, and the cost of health care services [26, 27], minimizing these barriers is essential to better leverage these critical tools in managing food insecurity.

Additional coping strategies vary on an individual and household level and play an important role in food insecurity management. Many food-insecure individuals report using social networks and financial strategies to alleviate the pressures of food insecurity [28]. Some of these strategies can contribute to the disruptive effect of food insecurity on health. For instance, food-insecure patients at an urban medical center report forgoing medical care to manage the financial stress of food insecurity [29].

Further, the usage of coping strategies has also been found to vary by ethnic and racial groups. A 2018 systematic review has examined strategies used by African American, American Indian, Hispanic, and Caucasian parents to cope with food insecurity. While the majority of ethnic and racial groups report using similar coping strategies (e.g., public or private assistance, nutrition-related strategies, financial-related strategies, and social networks), there are subtle differences in how these different groups employ these coping strategies [28]. For example, stigma has been noted as a barrier to the use of public assistance program among African American parents. Immigration has been noted as a barrier to the use of public assistance program among Hispanic parents. Therefore, differences in food insecurity management among racial or ethnic minoritized populations are of special interest in order to support the development and implementation of culturally relevant policies and community-based programs targeting food insecurity. Of note, this 2018 review did not include any study focusing on Asian Americans and food insecurity coping strategies [28].

Moreover, effective food insecurity management requires addressing the differences in the experience of food insecurity among racial and ethnic minority groups. Racial and ethnic disparities in food insecurity have been well-established for over 20 years [30–33]. For instance, national data on food insecurity trends shows that food insecurity among non-Hispanic Black and Hispanic households is more than twice that of non-Hispanic White households [2]. Various socioecological factors influence these racial and ethnic disparities in food insecurity. Social and economic disadvantages faced by minority groups play a critical role, as White households in the United States consistently have significantly higher median income, median wealth, and education than Black and Hispanic households [34]. However, racial and ethnic disparities in food insecurity persist even after these socioeconomic determinants are accounted for [35], and structural racism in the U.S. is increasingly being recognized as an important contributor to such disparities. Defined as the macro level systems, social forces, institutions, ideologies, and processes that interact with one another to generate and reinforce inequities among racial and ethnic minority groups [36], structural racism leads to increased food insecurity through both socioeconomic inequalities and other factors such as racial discrimination [37]. Additionally, upstream-level policies and practices create conditions that are not conducive to food security for these groups. For example, states with a higher concentration of minority groups are more likely to have stricter regulations and more severe punishments associated with food assistance programs [37], reducing enrollment and participation. Food deserts are also more common in neighborhoods with high concentrations of minority groups, which is often a result of segregation practices [38–40]. These disparities in food insecurity have been exacerbated by the COVID-19 pandemic, further necessitating the examination of food insecurity faced by different racial groups [41].

While a body of literature has focused on experiences of food insecurity in several racial and ethnic minority populations, this topic has been understudied among Asian Americans though they are one of the fastest growing demographic groups in the U.S. with an estimated population of 18.9 million [42]. In a literature review conducted by Maynard and colleagues that includes 33 studies on food insecurity among immigrants in the U.S., only two studies with food insecurity data of Asians were identified [43]. Similarly, a review of food insecurity coping strategies by racial and ethnic minority groups in the U.S. could not identify any studies that pertained to the Asian American population [28]. To date, while there are multiple literature reviews of experiences of food insecurity among other minority groups [44–46], no such review exists for food insecurity among Asian Americans.

Furthermore, there is a lack of disaggregated data among Asian Americans in many studies, and little is known about the heterogeneous experiences of food insecurity among different origin groups [47]. There are multiple possible reasons behind this discrepancy. The “model minority myth” that suggests that all Asian Americans achieve educational and occupational success may hinder the study of socioeconomic disadvantages among Asian Americans [48]. Additionally, the method and administration of national surveys may contribute to this lack of research. National surveys often aggregate Asian Americans into one category, which can hide any disparity that may exist among origin groups [47]. Many surveys are available in only English and Spanish, systematically excluding Asian individuals with limited English proficiency who may also experience higher food insecurity levels [49, 50].

Given these gaps in the literature, this review aims to establish what is known about the experiences of food insecurity and nutrition program participation in the Asian American population and among Asian origin groups and to suggest further research and policy action to better address food insecurity in this population. Because our goals are to broadly understand what has been written about the experiences of food insecurity among Asians living in the U.S and identifying literature gaps, a scoping review was deemed more appropriate for this purpose than a systematic review [51]. Our review is guided by the methodological framework proposed by Arksey and O’Malley [52] and refined and outlined by Levac and colleagues [53] and the Joanna Briggs Institute [54]. The protocol is reported based on the guidance provided by the Preferred Reporting Items for Systematic reviews and Meta-Analyses extension for Scoping Reviews (PRISMA-ScR) [55] (S1 Table).

## Materials and methods

### Stage 1: Identifying the research question

#### Objective

*Main research questions*. (Using the Population-Concept-Context framework [56])

What is known about the experiences of food insecurity among Asians living in the U.S?

Sub questions.

- How are Asian Americans defined or sampled in studies on food insecurity?
- Which origin groups of Asian Americans are experiencing the highest levels of food insecurity?
- How does food insecurity among Asian Americans compare to that in non-Asian populations?
- What are factors related to or influencing food insecurity among Asian Americans?
- What strategies are Asian Americans using to cope with or overcome food insecurity? What are factors related to or influencing the use of these strategies?

#### Protocol and registration

To our knowledge, no previous review protocol or review exists for this question. We have registered our protocol on the DigitalHub database hosted by Northwestern University [57].

### Stage 2: Identifying the relevant studies that will be included in the scoping review

The review team will work with a research librarian to develop an intensive search strategy. The search strategy will combine Medical Subject Headings (MeSH) terms and keywords for food insecurity and Asian Americans [58]. We first searched the Cochrane Database of Systematic Review, PubMed, and PROSPERO for possible existing reviews. We will apply and adapt the search strategy to Medline (Ovid), the Cochrane Library (Wiley), CINAHL Plus with Full Text (Ebsco), PsycINFO (Ebsco), and Scopus (Elsevier). We will exclude conference proceedings and limit the search studies published in English. Changes to the search will be documented. We will also review the reference lists of all included studies for additional relevant studies.

### Ovid Medline search

1. exp Food Insecurity/
2. exp Food Security/
3. exp Food Deserts/
4. exp Hunger/
5. exp Food Assistance/
*Food Services/
7. exp Food Supply/ and exp "Social Determinants of Health"/
8. exp Food Supply/ and exp Poverty/
9. exp "Diet, Food, and Nutrition"/ and exp "Social Determinants of Health"/
10. exp "Diet, Food, and Nutrition"/ and exp Poverty/
11. ("food aid" or "food bank" or "food banks" or "food desert*" or "food need*" or "food pantr*" or "food poor" or "food provision*" or "food stamp" or "food stamps" or "food sufficienc*" or hunger or "lack of food" or "SNAP Program*" or "soup kitchen*" or starvation or "wic program").ti,ab.
12. (food adj6 (access or assistance or depriv* or equity or hardship* or insecur* or insufficien* or program* or project* or poverty or scarcit* or scheme* or SDOH or "social determinates" or socioeconomic* or security or shortage* or shortfall* or suppl* or support* or school* or voucher* or wic or malnourishment or malnutrition or nutrition or overnutrition or undernutrition)).ti,ab.
13. ((breakfast* or dinner* or lunch* or meal* or nutrition* or supper*) adj6 (assistance or program* or project* or scheme* or support or school or voucher*)).ti,ab.
14. ("women infants and children" adj2 program).ti,ab.
15. 1 or 2 or 3 or 4 or 5 or 6 or 7 or 8 or 9 or 10 or 11 or 12 or 13 or 14
16. exp Asian Americans/
17. ((Asian* or Japanese or Korean* or Chinese or Bangladeshi or Bhutanese or Cambodian* or Filipino* or Hmong* Nepali or Nepalese or Pakistani* or Singaporean* or Sri Lankan* or Sinhalese or Taiwanese or Thai or Thais or Vietnamese or "from Asia" or "from Bangladesh" or "from Bhutan" or "from Cambodia" or "from China" or "from India" or "from Japan" or "from Korea" or "from Nepal" or "from Pakistan" or "from the Philippines" or "from Singapore" or "from Sri Lanka" or "from Taiwan" or "from Vietnam") adj6 (American* or "in the US" or "in the USA" or "in America" or "in the U.S." or "in the U.S.A." or "in the United States" or "US city" or "U.S. city" or "US cities" or "U.S. cities" or "US state" or "U.S. state" or "US states" or "U.S. states")).ti,ab.
18. ((Indian or Indians) adj6 (American* or "in the US" or "in the USA" or "in America" or "in the U.S." or "in the U.S.A." or "in the United States" or "US city" or "U.S. city" or "US cities" or "U.S. cities" or "US state" or "U.S. state" or "US states" or "U.S. states")).ti,ab. not (exp "American Indians or Alaska Natives"/ or "Native American*".ti,ab. or "American Indian*".ti,ab.)
19. (Asian* or Japanese or Korean* or Chinese or Bangladeshi or Bhutanese or Cambodian* or Filipino* or Hmong* Nepali or Nepalese or Pakistani* or Singaporean* or Sri Lankan* or Sinhalese or Taiwanese or Thai or Thais or Vietnamese or "from Asia" or "from Bangladesh" or "from Bhutan" or "from Cambodia" or "from China" or "from India" or "from Japan" or "from Korea" or "from Nepal" or "from Pakistan" or "from the Philippines" or "from Singapore" or "from Sri Lanka" or "from Taiwan" or "from Vietnam").ti,ab. and (American* or "U.S." or "in the US" or "in the USA" or "in America" or "in the U.S.A." or "in the United States" or "US city" or "US cities" or "US state" or "US states" or "of USA" or "of U.S.A.").ti.
20. ((Indian or Indians) and (American* or "U.S." or "in the US" or "in the USA" or "in America" or "in the U.S.A." or "in the United States" or "US city" or "US cities" or "US state" or "US states" or "of USA" or "of U.S.A.")).ti. not (exp "American Indians or Alaska Natives"/ or "Native American*".ti,ab. or "American Indian*".ti,ab.)
21. 16 or 17 or 18 or 19 or 20
22. 15 and 21
23. 22 not clinical conference.pt.
24. limit 23 to English language

### Stage 3: Selecting the studies

#### Eligibility (inclusion and exclusion) criteria

An article will be included if it

1. Was published in the English language
2. Is a peer-reviewed research manuscript and reports primary research findings from analyses
3. Describes food insecurity or strategies to cope with food insecurity among individuals of Asian origins living in the U.S.

An article will be excluded if it

1. Is a book, conference proceedings, or grey literature (e.g., thesis or dissertation)
2. Is a commentary, editorial, or opinion piece without primary research data
3. Contains only research conducted outside of the U.S.
4. Includes Asians in the sample but does not provide separate data on food insecurity or strategies to cope with food insecurity among Asians
5. Describes only dietary changes or patterns but not food insecurity

We will include qualitative, quantitative, and mixed-methods studies. If more than one articles originate from a study and report similar statistics or data, the most relevant article will be chosen for review.

#### Selection of sources of evidence

All retrieved studies from the search will be imported to Covidence. Two or more reviewers will screen the titles and abstracts of studies independently based on the inclusion and exclusion criteria. Potentially relevant studies will be selected for full-text reviews. We will conduct a pilot screening of 20 titles and abstracts. Similarly, we will pilot screen 10% of articles selected for full-text reviews. Any disagreement will be resolved through a discussion between the reviewers and with an additional reviewer if needed.

### Stage 4: Charting the data

#### Data charting process and data items

The data extraction template will record information from the final articles chosen to be included in the review. Based on Arksey and O’Malley’s recommendation [52], the template will include items related to study author, year of publication, setting, study population, methodology and study design, categorization and sampling of Asians, food insecurity levels and measurements, factors related to or influencing food insecurity, coping strategies, and factors related to or influencing coping strategies. This template will be iteratively improved during the research process. One reviewer will independently complete data extraction for each study. Three additional reviewers will independently review the completed tables. Discrepancies will be resolved through team discussion.

### Stage 5: Collating, summarizing, and reporting the results

The screening and study selection process will be documented through the PRISMA flow diagram [59]. We will use the PRISMA 2020 flow diagram for new reviews which included searches of databases, registers, and other sources to present our data (S1 Fig). We will prepare a summary narrative that synthesizes the information across key themes. Data will also be presented through tables. The report will describe results in relation to the main research question and the sub-questions. We will also identify literature gaps and implications for future research.

### Patient and public involvement statement

Patients and the public will not be involved in the design, analysis, or reporting of this study, which only synthesizes information from publicly available publications.

### Ethics and dissemination

We are not collecting and analyzing primary data; rather, we are reviewing and extracting information from already published studies. Therefore, ethical approval is not required. We will disseminate study results through peer-reviewed publications and conference presentations.

## Discussion

Food insecurity is an important social determinant of health linked to multiple individual and societal outcomes. The COVID-19 pandemic has exacerbated food insecurity in the United States [60, 61], warranting further examination to inform policy and practices that can better address food insecurity in general and close the gap in racial and ethnic disparities in food insecurity in particular. The aim of this review is to explore and synthesize existing evidence on food insecurity and the use of nutrition assistance programs among Asian Americans. We expect to identify possible disparities in food insecurity, important health-correlates of food insecurity, and different patterns of nutrition assistance program participation that can have broad implications for how food insecurity is addressed moving forward. The identification of disparities in food insecurity levels among Asian origin groups can play a crucial role in informing policy decisions aimed at extending and removing barriers to food assistance programs. Understanding the health-related impacts of food insecurity among Asian Americans can also reveal important implications and create policy opportunities to address disparities in these communities. By analyzing patterns of participation in nutrition assistance programs, we can identify specific obstacles to access and recommend changes to outreach as well as the administration and structure of these programs to promote equity in program uptake. Moreover, this review aims to evaluate the robustness of the current literature on food insecurity among Asian Americans, to identify gaps in knowledge and provide insights for future research in this area. Findings of this review will be invaluable in informing evidence-based policy decisions and practices that can address food insecurity in Asian American communities. This review will provide an overview of the evidence relevant to food insecurity in the Asian American population that will be of interest to researchers and practitioners concerned with food insecurity. We plan to disseminate the results through a peer-reviewed manuscript and conference presentations.

## Supporting information

S1 Table PRISMA-P (Preferred Reporting Items for Systematic review and Meta-Analysis Protocols) 2015 checklist: Recommended items to address in a systematic review protocol. (DOC)

S1 Fig PRISMA 2020 flow diagram for new systematic reviews which included searches of databases, registers and other sources. (DOCX)

## References

[1] USDA ERS—Measurement. [cited 5 Aug 2022]. Available: https://www.ers.usda.gov/topics/food-nutrition-assistance/food-security-in-the-u-s/measurement/

[2] Coleman-Jensen A, Rabbitt MP, Gregory CA, Singh A. Household Food Security in the United States in 2020. U.S. Department of Agriculture Economic Research Service; 2021 Sep. Report No.: 298. Available: https://www.ers.usda.gov/webdocs/publications/102076/err-298.pdf?v=2386

[3] USDA ERS—Food Security and Nutrition Assistance. [cited 5 Aug 2022]. Available: https://www.ers.usda.gov/data-products/ag-and-food-statistics-charting-the-essentials/food-security-and-nutrition-assistance/

[4] Coleman-Jensen A. Household Food Security in the United States in 2021. 2021.

[5] Gundersen C, Ziliak JP. Food Insecurity And Health Outcomes. Health Affairs. 2015;34: 1830–1839. doi: 10.1377/hlthaff.2015.0645 26526240

[6] Adams EJ, Grummer-Strawn L, Chavez G. Food Insecurity Is Associated with Increased Risk of Obesity in California Women. The Journal of Nutrition. 2003;133: 1070–1074. doi: 10.1093/jn/133.4.1070 12672921

[7] Ghose B, Tang S, Yaya S, Feng Z. Association between food insecurity and anemia among women of reproductive age. PeerJ. 2016;4: e1945. doi: 10.7717/peerj.1945 27168968 PMC4860303

[8] Thomas MMC, Miller DP, Morrissey TW. Food Insecurity and Child Health. Pediatrics. 2019;144: e20190397. doi: 10.1542/peds.2019-0397 31501236

[9] Whitaker RC, Phillips SM, Orzol SM. Food Insecurity and the Risks of Depression and Anxiety in Mothers and Behavior Problems in their Preschool-Aged Children. Pediatrics. 2006;118: e859–e868. doi: 10.1542/peds.2006-0239 16950971

[10] Heflin CM, Ziliak JP. Food Insufficiency, Food Stamp Participation, and Mental Health. Social Science Quarterly. 2008;89: 706–727. doi: 10.1111/j.1540-6237.2008.00556.x

[11] Casey P, Goolsby S, Berkowitz C, Frank D, Cook J, Cutts D, et al. Maternal Depression, Changing Public Assistance, Food Security, and Child Health Status. Pediatrics. 2004;113: 298–304. doi: 10.1542/peds.113.2.298 14754941

[12] Berkowitz SA, Seligman HK, Meigs JB, Basu S. Food Insecurity, Healthcare Utilization, and High Cost: A Longitudinal Cohort Study. Am J Manag Care. 2018;24: 399–404. 30222918 PMC6426124

[13] Jia J, Fung V, Meigs JB, Thorndike AN. Food Insecurity, Dietary Quality, and Health Care Utilization in Lower-Income Adults: A Cross-Sectional Study. Journal of the Academy of Nutrition and Dietetics. 2021;121: 2177–2186.e3. doi: 10.1016/j.jand.2021.06.001 34247978

[14] Hunger in America. Available: https://www.americanprogress.org/article/hunger-in-america/

[15] Brown ME, Antle JM, Backlund P, Carr ER, Easterling WE, Walsh MK, et al. Climate Change, Global Food Security, and the U.S. Food System. U.S. Global Change Research Program; 2015. doi: 10.7930/J0862DC7

[16] Bauer L. The COVID-19 crisis has already left too many children hungry in America. In: Brookings [Internet]. 6 May 2020 [cited 29 Apr 2023]. Available: https://www.brookings.edu/blog/up-front/2020/05/06/the-covid-19-crisis-has-already-left-too-many-children-hungry-in-america/

[17] Ben Hassen T, El Bilali H. Impacts of the Russia-Ukraine War on Global Food Security: Towards More Sustainable and Resilient Food Systems? Foods. 2022;11: 2301. doi: 10.3390/foods11152301 35954068 PMC9368568

[18] Hall L, Nchako C. A Closer Look at Who Benefits from SNAP: State-by-State Fact Sheets. In: Center on Budget and Policy Priorities [Internet]. [cited 21 Jul 2022]. Available: https://www.cbpp.org/research/food-assistance/a-closer-look-at-who-benefits-from-snap-state-by-state-fact-sheets

[19] Supplemental Nutrition Assistance Program (U.S.), Caswell JA, Yaktine AL, Institute of Medicine (U.S.), National Research Council (U.S.), National Research Council (U.S.), editors. Supplemental Nutrition Assistance Program: Examining the Evidence to Define Benefit Adequacy. Washington, D.C: National Academies Press; 2013. 24901188

[20] About WIC—WIC at a Glance | Food and Nutrition Service. [cited 5 Aug 2022]. Available: https://www.fns.usda.gov/wic/about-wic-glance

[21] Costanzo C. How Many Food Banks Are There? In: Food Bank News [Internet]. 8 Jan 2020 [cited 5 Aug 2022]. Available: https://foodbanknews.org/how-many-food-banks-are-there/

[22] December 2019 CPS Food Security Supplement Data File: Technical Documentation. U.S. Department of Agriculture Economic Research Service; 2020. Available: https://www.ers.usda.gov/webdocs/DataFiles/50764/techdoc2019.pdf?v=8379.6

[23] Martin KS, Cook JT, Rogers BL, Joseph HM. Public versus Private Food Assistance: Barriers to Participation Differ by Age and Ethnicity. Journal of Nutrition Education and Behavior. 2003;35: 249–254. doi: 10.1016/s1499-4046(06)60055-9 14521824

[24] Algert SJ, Reibel M, Renvall MJ. Barriers to Participation in the Food Stamp Program Among Food Pantry Clients in Los Angeles. Am J Public Health. 2006;96: 807–809. doi: 10.2105/AJPH.2005.066977 16571694 PMC1470578

[25] Dodds JM, Ahluwalia I, Baligh M. Experiences of Families Using Food Assistance and Welfare Programs in North Carolina: Perceived Barriers and Recommendations for Improvement. Journal of Nutrition Education. 1996;28: 101–108. doi: 10.1016/S0022-3182(96)70035-3

[26] Fox M, Hamilton W, Lin B-Hwan. Executive Summary of the Literature Review. Effects of Food Assistance and Nutrition Programs on Nutrition and Health. 2004.

[27] Gregory CA, Deb P. Does SNAP improve your health? Food Policy. 2015;50: 11–19. doi: 10.1016/j.foodpol.2014.09.010

[28] Kamdar N, Rozmus CL, Grimes DE, Meininger JC. Ethnic/Racial Comparisons in Strategies Parents Use to Cope with Food Insecurity: A Systematic Review of Published Research. J Immigrant Minority Health. 2019;21: 175–188. doi: 10.1007/s10903-018-0720-y 29549476

[29] Mayer VL, McDonough K, Seligman H, Mitra N, Long JA. Food insecurity, coping strategies and glucose control in low-income patients with diabetes. Public Health Nutr. 2016;19: 1103–1111. doi: 10.1017/S1368980015002323 26328922 PMC10270824

[30] Odoms-Young A, Bruce MA. Examining the Impact of Structural Racism on Food Insecurity: Implications for Addressing Racial/Ethnic Disparities. Family & Community Health. 2018;41: S3–S6. doi: 10.1097/FCH.0000000000000183 29461310 PMC5823283

[31] Morales DX, Morales SA, Beltran TF. Racial/Ethnic Disparities in Household Food Insecurity During the COVID-19 Pandemic: a Nationally Representative Study. J Racial and Ethnic Health Disparities. 2021;8: 1300–1314. doi: 10.1007/s40615-020-00892-7 33057998 PMC7556612

[32] Hernandez DC, Reesor LM, Murillo R. Food insecurity and adult overweight/obesity: Gender and race/ethnic disparities. Appetite. 2017;117: 373–378. doi: 10.1016/j.appet.2017.07.010 28739148

[33] Walker RJ, Garacci E, Dawson AZ, Williams JS, Ozieh M, Egede LE. Trends in Food Insecurity in the United States from 2011–2017: Disparities by Age, Sex, Race/Ethnicity, and Income. Population Health Management. 2021;24: 496–501. doi: 10.1089/pop.2020.0123 32941115 PMC8403212

[34] Williams DR, Priest N, Anderson NB. Understanding associations among race, socioeconomic status, and health: Patterns and prospects. Health Psychology. 2016;35: 407–411. doi: 10.1037/hea0000242 27018733 PMC4817358

[35] Myers AM, Painter MA. Food insecurity in the United States of America: an examination of race/ethnicity and nativity. Food Sec. 2017;9: 1419–1432. doi: 10.1007/s12571-017-0733-8

[36] Powell J. Structural Racism: Building upon the Insights of John Calmore. North Carolina Law Review. 2008;86: 791–816.

[37] Bowen S, Elliott S, Hardison‐Moody A. The structural roots of food insecurity: How racism is a fundamental cause of food insecurity. Sociology Compass. 2021;15. doi: 10.1111/soc4.12846

[38] Elsheikh E, Barhoum N. Structural Racialization and Food Insecurity in the United States. HAAS Institute for a Fair and Inclusive Society, University of California, Berkeley; 2013 Sep. Available: https://haasinstitute.berkeley.edu/sites/default/files/Structural%20Racialization%20%20%26%20Food%20Insecurity%20in%20the%20US-%28Final%29.pdf

[39] Cooksey Stowers K, Jiang Q, Atoloye A, Lucan S, Gans K. Racial Differences in Perceived Food Swamp and Food Desert Exposure and Disparities in Self-Reported Dietary Habits. IJERPH. 2020;17: 7143. doi: 10.3390/ijerph17197143 33003573 PMC7579470

[40] Moore NY. The South Side: a portrait of Chicago and American segregation. First edition. New York: St. Martin’s Press; 2016.

[41] Lauren BN, Silver ER, Faye AS, Rogers AM, Woo-Baidal JA, Ozanne EM, et al. Predictors of households at risk for food insecurity in the United States during the COVID-19 pandemic. Public Health Nutr. 2021;24: 3929–3936. doi: 10.1017/S1368980021000355 33500018 PMC8207551

[42] Budiman A, Ruiz NG. Asian Americans are the fastest-growing racial or ethnic group in the U.S. In: Pew Research Center [Internet]. [cited 24 Aug 2022]. Available: https://www.pewresearch.org/fact-tank/2021/04/09/asian-americans-are-the-fastest-growing-racial-or-ethnic-group-in-the-u-s/

[43] Maynard M, Dean J, Rodriguez PI, Sriranganathan G, Qutub M, Kirkpatrick SI. The Experience of Food Insecurity Among Immigrants: a Scoping Review. Int Migration & Integration. 2019;20: 375–417. doi: 10.1007/s12134-018-0613-x

[44] Nikolaus CJ, Johnson S, Benally T, Maudrie T, Henderson A, Nelson K, et al. Food Insecurity among American Indian and Alaska Native People: A Scoping Review to Inform Future Research and Policy Needs. Advances in Nutrition. 2022; nmac008. doi: 10.1093/advances/nmac008 35092417 PMC9526849

[45] Gingell T, Murray K, Correa-Velez I, Gallegos D. Determinants of food security among people from refugee backgrounds resettled in high-income countries: A systematic review and thematic synthesis. Jalal S, editor. PLoS ONE. 2022;17: e0268830. doi: 10.1371/journal.pone.0268830 35653308 PMC9162305

[46] Greene M, Houghtaling B, Sadeghzadeh C, De Marco M, Bryant D, Morgan R, et al. Nutrition interventions addressing structural racism: a scoping review. Nutr Res Rev. 2022; 1–20. doi: 10.1017/S0954422422000014 35022096

[47] Becerra M, Mshigeni S, Becerra B. The Overlooked Burden of Food Insecurity among Asian Americans: Results from the California Health Interview Survey. IJERPH. 2018;15: 1684. doi: 10.3390/ijerph15081684 30087306 PMC6121379

[48] Yi V, Museus SD. Model Minority Myth. In: Smith AD, Hou X, Stone J, Dennis R, Rizova P, editors. The Wiley Blackwell Encyclopedia of Race, Ethnicity, and Nationalism. Oxford, UK: John Wiley & Sons, Ltd; 2015. pp. 1–2. doi: 10.1002/9781118663202.wberen528

[49] Rothwell CJ, Madans JH, Porter KS. National Health and Nutrition Examination Survey: Plan and Operations, 1999–2010. U.S. Department of Health and Human Services, Center for Disease Control and Prevention, National Center for Health Statistics; 2013 Aug. Report No.: 56. Available: https://www.cdc.gov/nchs/data/series/sr_01/sr01_056.pdf

[50] CDC—BRFSS—Questionnaires. 8 Jun 2022 [cited 13 Aug 2022]. Available: https://www.cdc.gov/brfss/questionnaires/index.htm

[51] Munn Z, Peters MDJ, Stern C, Tufanaru C, McArthur A, Aromataris E. Systematic review or scoping review? Guidance for authors when choosing between a systematic or scoping review approach. BMC Med Res Methodol. 2018;18: 143. doi: 10.1186/s12874-018-0611-x 30453902 PMC6245623

[52] Arksey H, O’Malley L. Scoping studies: towards a methodological framework. International Journal of Social Research Methodology. 2005;8: 19–32. doi: 10.1080/1364557032000119616

[53] Levac D, Colquhoun H, O’Brien KK. Scoping studies: advancing the methodology. Implementation Sci. 2010;5: 69. doi: 10.1186/1748-5908-5-69 20854677 PMC2954944

[54] Peters M, Godfrey C, McInerney P, Munn Z, Trico A, Khalil H. Chapter 11: Scoping Reviews. In: Aromataris E, Munn Z, editors. JBI Manual for Evidence Synthesis. JBI; 2020. doi: 10.46658/JBIMES-20-12

[55] Tricco AC, Lillie E, Zarin W, O’Brien KK, Colquhoun H, Levac D, et al. PRISMA Extension for Scoping Reviews (PRISMA-ScR): Checklist and Explanation. Ann Intern Med. 2018;169: 467–473. doi: 10.7326/M18-0850 30178033

[56] Delaney L. Guides: Scoping Reviews: Apply PCC. [cited 24 Aug 2022]. Available: https://guides.library.unisa.edu.au/ScopingReviews/ApplyPCC

[57] Ro Suji. Food Insecurity among Asian Americans: A Scoping Review Protocol. [cited 24 Aug 2022]. doi: 10.18131/G3-BGNB-VG80 PMC10317216 37399223

[58] Medical Subject Headings—Home Page. U.S. National Library of Medicine; [cited 13 Sep 2022]. Available: https://www.nlm.nih.gov/mesh/meshhome.html

[59] PRISMA. [cited 24 Aug 2022]. Available: https://prisma-statement.org/PRISMAStatement/FlowDiagram

[60] Wolfson JA, Leung CW. Food Insecurity During COVID-19: An Acute Crisis With Long-Term Health Implications. Am J Public Health. 2020;110: 1763–1765. doi: 10.2105/AJPH.2020.305953 32970451 PMC7662000

[61] Vu M, Makelarski JA, Winslow VA, Christmas MM, Haider S, Lee NK, et al. Racial and Ethnic Disparities in Health-Related Socioeconomic Risks During the Early COVID-19 Pandemic: A National Survey of U.S. Women. Journal of Women’s Health. 2021;30: 1375–1385. doi: 10.1089/jwh.2021.0230 34529520 PMC8590141

