# Supplementary material for: Food insecurity among Asian Americans: A scoping review protocol
Source: PLoS One. 2023 Jul 3;18(7):e0287895. doi: 10.1371/journal.pone.0287895 (PMC10317216; doi:10.1371/journal.pone.0287895)
Supplement: S1 Fig — (DOCX) [file pone.0287895.s002.docx]

**Identification of studies via other methods**

**Identification of studies via databases and registers**

Records identified from**:

Other database (n = )

Records removed *before screening*:

Duplicate records removed (n = )

Records marked as ineligible by automation tools (n = )

Records identified from*:

Medline (n = )

Cochrane (n = )

Scopus (n = )

CINAHL (n = )

PsycInfo (n = )

**Identification**

Records screened

(n = )

Records excluded

(n = )

Reports not retrieved

(n = )

Reports sought for retrieval

(n = )

Reports sought for retrieval

(n = )

Reports not retrieved

(n = )

**Screening**

Reports excluded:

Book, conference proceeding, or grey literature (n = )

Commentary, editorial, or opinion piece without primary research data (n = )

Contains only research conducted outside of the U.S. (n = )

Includes Asians in the sample but no separate data on food insecurity or coping strategies (n = )

Only dietary changes or patterns but not food insecurity (n =)

etc.

Reports assessed for eligibility

(n = )

Reports excluded:

(n = )

Reports assessed for eligibility

(n = )

Studies included in review

(n = )

Reports of included studies

(n = )

**Included**

*From:*  Page MJ, McKenzie JE, Bossuyt PM, Boutron I, Hoffmann TC, Mulrow CD, et al. The PRISMA 2020 statement: an updated guideline for reporting systematic reviews. BMJ 2021;372:n71. doi: 10.1136/bmj.n71. For more information, visit: <http://www.prisma-statement.org/>
